# Increased LL37 in psoriasis and other inflammatory disorders promotes LDL uptake and atherosclerosis

**DOI:** 10.1172/JCI172578

**Published:** 2024-03-01

**Authors:** Yoshiyuki Nakamura, Nikhil N. Kulkarni, Toshiya Takahashi, Haleh Alimohamadi, Tatsuya Dokoshi, Edward Liu, Michael Shia, Tomofumi Numata, Elizabeth W.C. Luo, Adrian F. Gombart, Xiaohong Yang, Patrick Secrest, Philip L.S.M. Gordts, Sotirios Tsimikas, Gerard C.L. Wong, Richard L. Gallo

**Affiliations:** 1 Department of Dermatology and; 2 Department of Bioengineering, UCLA, Los Angeles, California, USA.; 3 Linus Pauling Institute, Department of Biochemistry and Biophysics, Oregon State University, Corvallis, Oregon, USA.; 4 Division of Cardiovascular Diseases,; 5 Department of Medicine, Division of Endocrinology and Metabolism, and; 6 Glycobiology Research and Training Center, UCSD, La Jolla, California, USA.

**Keywords:** Cardiology, Dermatology, Atherosclerosis, Innate immunity, Skin

## Abstract

Patients with chronic inflammatory disorders such as psoriasis have an increased risk of cardiovascular disease and elevated levels of LL37, a cathelicidin host defense peptide that has both antimicrobial and proinflammatory properties. To explore whether LL37 could contribute to the risk of heart disease, we examined its effects on lipoprotein metabolism and show that LL37 enhanced LDL uptake in macrophages through the LDL receptor (LDLR), scavenger receptor class B member 1 (SR-B1), and CD36. This interaction led to increased cytosolic cholesterol in macrophages and changes in expression of lipid metabolism genes consistent with increased cholesterol uptake. Structure-function analysis and synchrotron small-angle x-ray scattering showed structural determinants of the LL37-LDL complex that underlie its ability to bind its receptors and promote uptake. This function of LDL uptake is unique to cathelicidins from humans and some primates and was not observed with cathelicidins from mice or rabbits. Notably, *Apoe^–/–^* mice expressing LL37 developed larger atheroma plaques than did control mice, and a positive correlation between plasma LL37 and oxidized phospholipid on apolipoprotein B (OxPL-apoB) levels was observed in individuals with cardiovascular disease. These findings provide evidence that LDL uptake can be increased via interaction with LL37 and may explain the increased risk of cardiovascular disease associated with chronic inflammatory disorders.

## Abstract 2

The excess production of LL37 in human inflammatory skin disorders such as psoriasis enhances LDL uptake and may increase the risk of cardiovascular disease.

## Graphical abstract

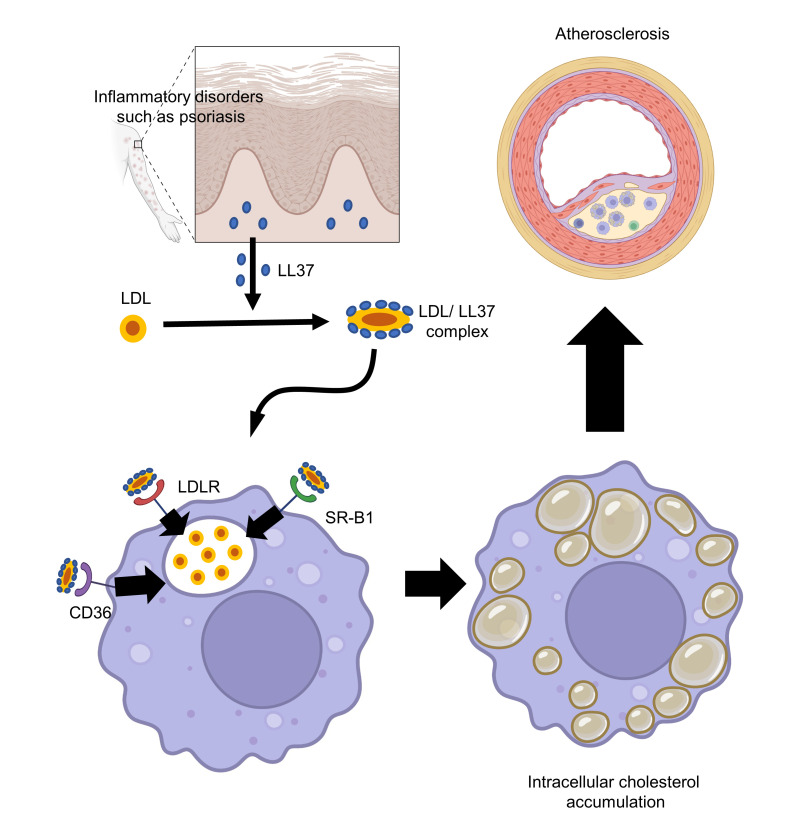

## Introduction

Atherosclerosis is characterized by lipid accumulation and local inflammation in the arterial vessel wall and is a major cause of cardiovascular diseases such as myocardial infarction and peripheral arterial disease (1). Although many types of cells are involved in the uptake of lipid and formation of the atheroma plaque, macrophage-derived foam cells are thought to play a central role (2). Well-known risk factors for the development of atherosclerosis include hypercholesterolemia, obesity, hypertension, and smoking. Furthermore, multiple studies have also demonstrated that some disorders of chronic skin inflammation such as psoriasis and rosacea are independent risk factors for cardiovascular comorbidities (3–6). Indeed, the severity of psoriasis positively correlates with a higher likelihood of cardiovascular comorbidities (7). In a large population-based cohort study, the HR of the risk of cardiovascular mortality in patients with severe psoriasis after adjustment was made for major cardiovascular risk factors was 1.57 (95% CI 1.26–1.96), which was even higher than that observed with hypertension and smoking (6). In addition to disorders of skin inflammation, chronic inflammatory disorders of other organ systems such as inflammatory bowel diseases (IBD) and rheumatoid arthritis (RA) also have an increased risk of cardiovascular comorbidities (8–14). Despite the high prevalence of these inflammatory diseases and their clinical effect on cardiovascular disease, mechanistic insights into why such chronic inflammation is associated with an increased risk of atherosclerosis remains elusive.

One common characteristic of inflammatory skin diseases is the increased expression of antimicrobial peptides (AMPs) such as cathelicidin. Cathelicidins are an evolutionarily ancient gene family that acts as an important effector molecule for host defense and inflammation (15). The precursor domain of cathelicidin pro-proteins is conserved, but active mature peptides are highly variable between species. The only human cathelicidin gene, called *CAMP*, is produced by many cell types, including neutrophils, epithelial cells, and preadipocytes, and encodes the mature peptide LL37, a 37-residue, cationic, amphipathic and α-helical peptide (15). LL37 is released from its precursor protein hCAP18 by proteolytic cleavage (16). In addition to its antimicrobial activity, LL37 also triggers inflammation by activating inflammatory signaling events in keratinocytes, endothelial cells, and macrophages (17–19). This proinflammatory activity occurs as a result of several properties of this peptide including the capacity to activate G-protein–coupled cell-surface receptors and facilitate the uptake of nucleic acids to trigger intercellular pattern recognition receptor signaling (17, 20, 21). Although the expression of cathelicidin is strictly regulated, its expression is greatly induced during inflammatory conditions. In particular, previous studies have demonstrated that serum LL37/hCAP18 levels were significantly higher in patients with psoriasis than in healthy individuals (22–24). Although a validated clinical assay for serum LL37/ hCAP18 concentrations does not yet exist, multiple studies using different assay techniques have reported that this AMP is higher in patients with skin inflammation. For example, the mean values of LL37/ hCAP18 in 1 study were reported as 970 ng/mL in sera from patients with psoriasis and 741 ng/mL in normal sera (22), whereas another study reported LL37/ hCAP18 levels in seras from patients with psoriasis as 106.3 ng/mL compared with 3.8 ng/mL in sera from healthy controls (24). In addition to psoriasis, patients with chronic inflammation such as rosacea, IBD, and RA have also been reported to have elevated serum levels of LL37 compared with healthy individuals (25–28). LL37 has also been observed to accumulate in atheroma plaques (29) and bind to lipoproteins (30–32). On the basis of these observations and on correlations between diseases with elevated LL37 and coronary artery disease, it has been hypothesized that LL37 could contribute to the development of atherosclerosis.

In this study, we sought to determine whether LL37 could actively contribute to the development of atheroma and therefore provide a potential explanation for the association between inflammatory disorders that feature high levels of LL37 and cardiovascular diseases. We show that LL37 could increase the uptake of lipid particles such as LDL and that LL37 facilitated the development of atherosclerosis in mice. These observations uncover a previously unknown pathway for inducing increased lipoprotein uptake and may explain why the chronic inflammatory disorders that feature elevated circulating levels of LL37 confer an increased risk of cardiovascular disease.

## Results

### LL37 promotes an increased uptake of LDL.

**Figure 1:**
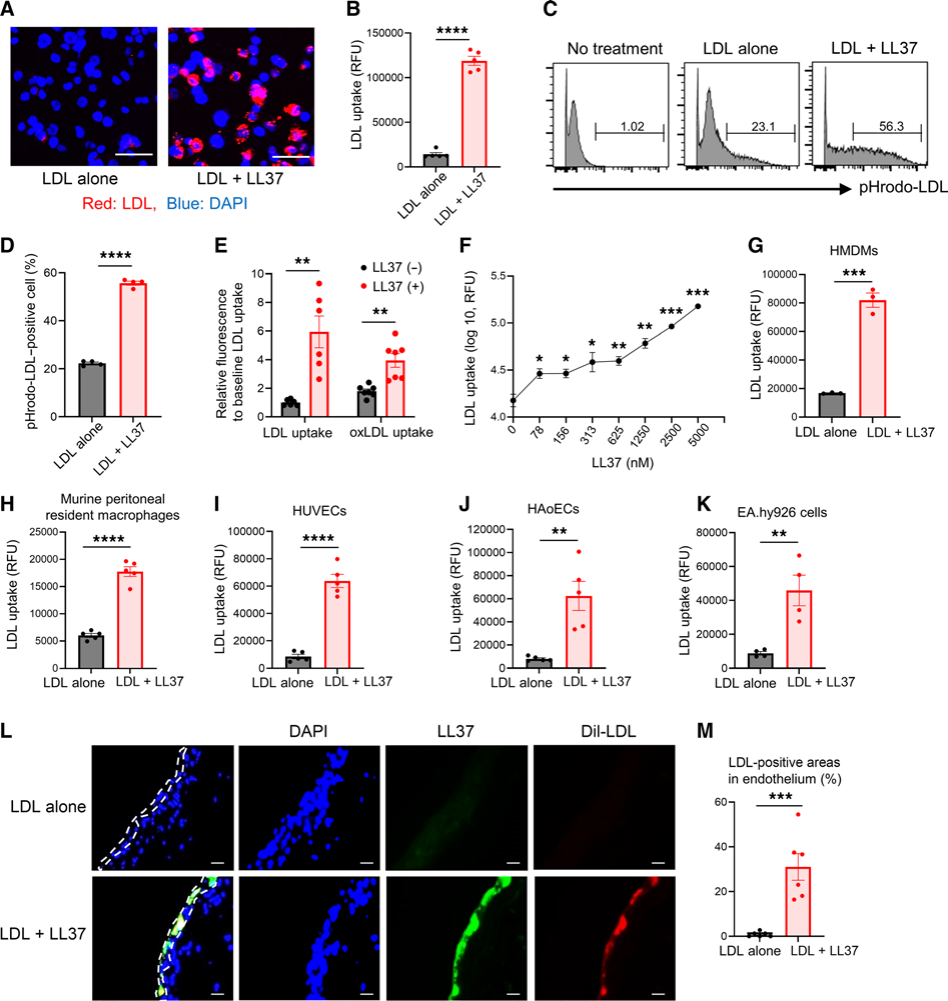
LL37 promotes LDL entry into cells. (**A**) Visualization of pHrodo-LDL in THP-1 macrophages in the absence or presence of LL37. (**B**) Total fluorescence of pHrodo-LDL in THP-1 macrophages treated as in **A** (*n* = 5 per group). (**C**) FACS analysis and (**D**) proportion of CD45^+^ pHrodo-LDL+ THP-1 cells after treatment with LL37 (*n* = 6 per group). (**E**) Comparison of pHrodo-LDL or pHrodo-oxLDL uptake in the presence or absence of LL37 in THP-1 macrophages (*n* = 6–7 per group). (**F**) Dose-dependent uptake of pHrodo-LDL at the indicated concentrations of LL37 in THP-1 macrophages (*n* = 4 per concentration). (**G** and **H**) Uptake of pHrodo-LDL into HMDMs (*n* = 3 per group) (**G**), primary murine peritoneal macrophages (*n* = 5 per group) (**H**), HUVECs (*n* = 5 per group) (**I**), HAoECs (*n* = 5 per group) (**J**), and EA.hy926 endothelial cells (*n* = 4 per group) (**K**) treated with LL37. (**L**) Representative images of Dil-LDL uptake (red) and LL37 (green) in mouse aortas treated with LL37. White dotted lines outline the endothelial layer. (**M**) Proportion of positive fluorescence areas for Dil-LDL in aortic endothelium in presence and absence of LL37. Scales bars: 50 μm (**A** and **L**). Data indicate mean ± SEM. *P < 0.05, **P < 0.01, ***P < 0.001, and ****P < 0.0001, by 2-tailed Student’s *t* test (Student’s *t* test relative to no treatment in **F**).

The uptake of LDL and modified LDL by macrophages is a crucial step in the development of atherosclerosis (33). LL37 promotes entry of nucleic acids such as U1-RNA into the cytosol via scavenger receptors (17, 18, 34, 35). Since binding of LDL to cell-surface scavenger receptors such as SR-B1 facilitates its uptake (36), we hypothesized that LL37 may also promote the entry of LDL particles. To test this hypothesis, pHrodo-labeled LDL, which is only visible after cell internalization, was added to THP-1 macrophages in the presence or absence of LL37. Under these conditions, we observed that LL37 increased cytosolic LDL accumulation (Figure 1, A–D). LL37 also increased the uptake of oxidized LDL (oxLDL), VLDL, and HDL, but the relative increase was greatest for LDL (Figure 1E and Supplemental Figure 1A; supplemental material available online with this article; https://doi.org/10.1172/JCI172578DS1). The uptake of LDL into THP-1 macrophages was dependent on the LL37 concentration, with a minimum LL37 concentration of 78 nM required for LDL uptake (Figure 1F). LL37 also promoted LDL uptake into human monocyte–derived macrophages (HMDMs) and primary murine peritoneal tissue–resident macrophages (Figure 1, G and H). LDL uptake was also enhanced in endothelial cells, including human umbilical vein endothelial cells (HUVECs), human aortic endothelial cells (HAoECs), and EA.hy926 cells, by LL37 (Figure 1, I–K), and mouse aortas cultured ex situ with Dil-LDL (1,1′-dioctadecyl-3,3,3′,3′-tetramethylindocarbocyanine perchlorate-low density lipoprotein–LDL) further demonstrated that addition of LL37 increased LDL accumulation in the aortic endothelium (Figure 1, L and M). Notably, the fluorescent signal from LL37 overlapped with the signal from LDL, suggesting that LL37 might form complexes with LDL (Figure 1L). These results suggested that the mature human cathelicidin peptide LL37 could promote LDL uptake into macrophages and endothelial cells.

### The capacity to promote uptake of LDL is not present in all AMPs.

**Figure 2:**
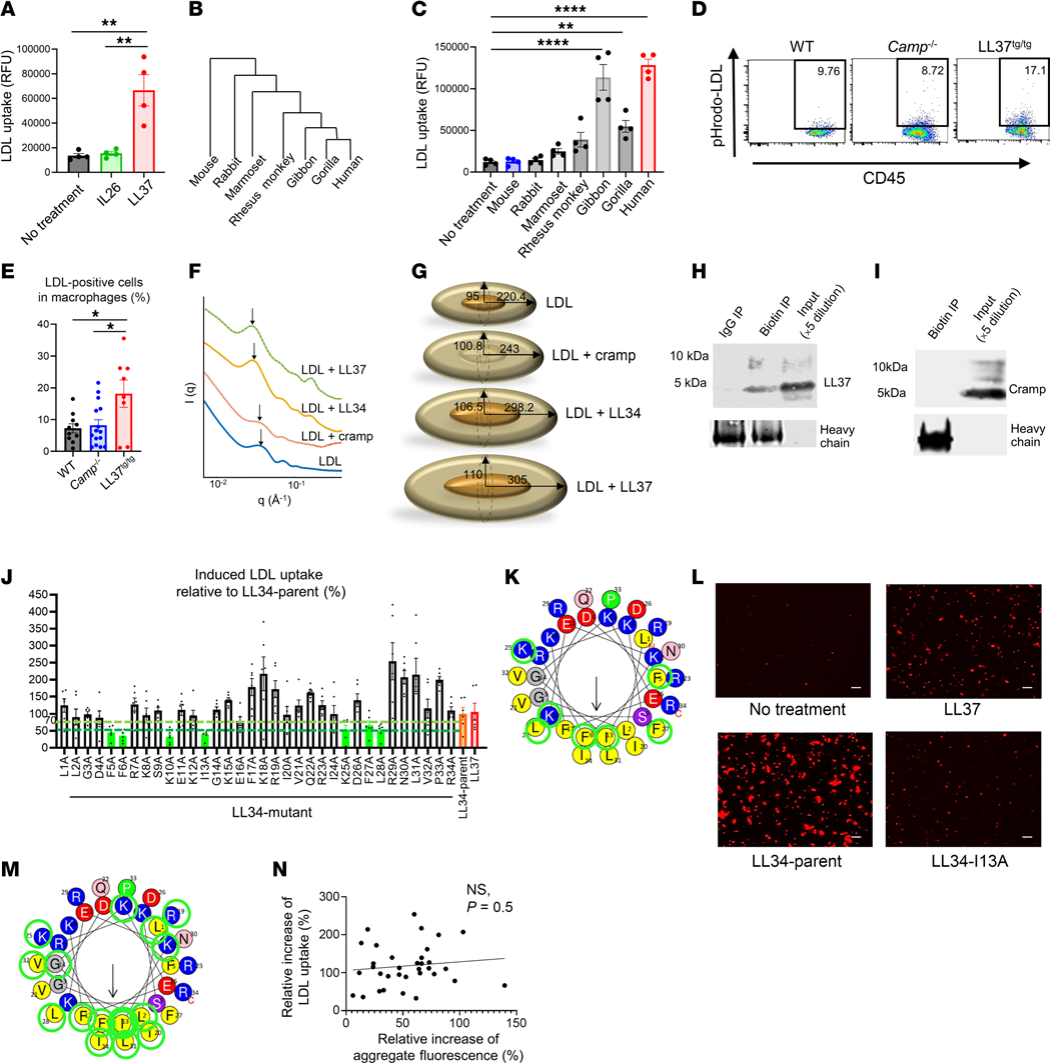
Sequence elements of LL37 that promote uptake of LDL. (**A**) Uptake of pHrodo-LDL into THP-1 cells treated with IL-26 or LL37 (*n* = 4 per group). (**B**) Phylogenic tree of the cathelicidin gene family. (**C**) pHrodo-LDL uptake into THP-1 cells treated with cathelicidin peptides from the indicated species (*n* = 4 in each group). (**D** and **E**) FACS analysis of pHrodo-LDL^+^ cells in CD45^+^CD11b^+^F4/80^+^ gated macrophages following peritoneal injection of pHrodo-LDL (*n* = 8–15 in each group). (**F**) SAXS profile of LDL incubated with LL37, LL34, or Cramp at a P/L ratio of 3:35. Arrows show the location of the first peak in the intensity profile, *q*_peak_-LDL = 0.036Å^–1^, *q*_peak_-LDL-Cramp = 0.032Å^–1^, *q*_peak_-LDL-LL34 = 0.029Å^–1^, and *q*_peak_-LDL-LL37 = 0.028Å^–1^. (**G**) Schematic of the size and structure of the LDL particle and complexes based on the fitted models of concentric core-shell ellipsoids to the SAXS spectra. The dimensions are given in angstrom. (**H** and **I**) Coimmunoprecipitation (IP) of biotinylated LDL and detection with anti-LL37 (**H**) or anti-Cramp (**I**) antibodies. (**J**) pHrodo-LDL uptake into THP-1 cells after addition of LL37, LL34, or LL34 with alanine substitutions at positions 1–34 (LL34 L1A-R34A) (*n* = 6 per group). (**K**) Helical wheel plot of LL34 with green circles indicating substitutions resulting in more than a 30% decrease in LDL uptake compared with parent LL34 peptide. (**L**) Representative immunofluorescence study of Dil-LDL aggregate cultured with LL37, LL34, or LL34-I13A. Scale bars: 20 μm. (**M**) Helical wheel plot of LL34, in which green circles indicate the position where alanine substitution resulted in more than a 50% decrease in aggregate fluorescence. (**N**) Linear regression analysis for associations between LDL uptake and fluorescence of LDL aggregate induced by the LL34 mutant peptides. Data indicate the mean ± SEM; *P < 0.05, **P < 0.01, and ****P < 0.0001, by Dunnett’s test (**C**) or 1-way ANOVA multiple-comparison test (**A** and **E**).

To further understand the importance of the observation that LL37 could increase LDL within cells, we next compared this function with other peptides that provide host defense and are increased during inflammation. Several naturally occurring peptides can alter membrane properties and have antimicrobial activity, and some, like IL26, have common activities with LL37 to promote entry of DNA into the cytosol (37, 38). IL-26 did not show the capacity to promote LDL uptake in THP-1 cells (Figure 2A). A comparison of cathelicidin AMPs that are present in different mammalian species (39) also showed that not all AMPs can increase LDL uptake. The cathelicidin mature peptide from great apes (hominidae) has the highest similarity to human LL37, followed by gibbon (hylobatidae), Old World monkey (i.e., rhesus macaque), New World monkey (i.e., marmosets), rabbit, and mouse (Figure 2B) (40, 41). These peptides have a similar capacity to kill bacteria, and some show a similar ability to increase inflammatory gene expression (40, 42–47). Cathelicidin peptides from humans, gorillas, and gibbons promoted a significant level of LDL uptake in THP-1 cells, whereas the peptides from more distant evolutionary species did not (Figure 2C). Thus, the antimicrobial activity of cathelicidin peptides did not correlate with the capacity to increase LDL uptake.

Having observed in vitro that the mouse cathelicidin mature peptide did not increase LDL uptake, we next evaluated the potential of LL37 to promote LDL uptake in vivo by testing humanized transgenic mice carrying the human *CAMP* gene (LL37^tg/tg^) (18, 48). Macrophages were recruited into the peritoneal cavity by thioglycolate injection followed by injection of pHrodo-LDL into the peritoneum 48 hours after thioglycolate. FACS analysis of peritoneal cells collected 18 hours after the pHrodo-LDL injection showed elevated LDL uptake in macrophages from LL37^tg/tg^ mice compared with *Camp^–/–^* or WT mice (Figure 2, D and E). Macrophages from *Camp^–/–^* mice had similar levels of LDL uptake compared with macrophages from WT mice (Figure 2, D and E). These results further show that human LL37, but not the mature mouse cathelicidin, promoted LDL uptake.

### Structural elements of LL37 that promote uptake of LDL.

We next sought to understand the characteristics of LL37 that increase LDL uptake. Since LL37 fluorescence overlapped with LDL signals in cultured primary endothelial cells (Figure 1L) and LL37 has been previously shown to form complexes with nucleic acids such as dsDNA and U1-RNA (17, 18, 34, 35) as well as lipoproteins (30–32), we hypothesized that LL37 might form a complex with LDL that would facilitate LDL uptake into cells. To investigate the nanoscale characteristics of the interactions between LDL particles and LL37 and other cathelicidin peptides, we used high-resolution synchrotron small-angle x-ray scattering (SAXS) and quantitatively analyzed whether LDL is remodeled by interactions with LL37, LL34, or mouse Cramp. LL34 is a variant of LL37 that has been truncated by 3 amino acids at the carboxyl terminus but maintains properties similar to those of LL37 and therefore served as a positive control (49). The mouse cathelicidin mature peptide Cramp has a similar peptide charge, amphipathic α-helical structure, and antimicrobial potency (15, 43) and served as a negative control, given our prior observation that it did not induce LDL uptake. The SAXS data for LDL revealed an oscillatory form factor that was similar to what has been observed in previous studies (50) (blue line in Figure 2F). Upon exposure of LDL particles to LL37 and LL34 (peptide-to-lipid [P/L] molar ratio = 3:35), we observed a substantial shift in the oscillatory features toward smaller *q* values, which suggests an increase in the size of the LDL particle. For example, the oscillation feature peaked at *q* = 0.036 Å^–1^ for LDL shifts to *q* = 0.028 Å^–1^ and *q* = 0.029 Å^–1^ for LDL complexes with LL37 and LL34, respectively (Figure 2F). However, the corresponding feature for the LDL complex with Cramp at the same P/L ratio exhibited a slight shift to a value of *q* = 0.032 Å^–1^ (Figure 2F). This implies that LDL interactions with LL37 and LL34 were similar, in contrast to those with Cramp.

To predict the LL37-induced geometric change in the LDL particles and the LL34-LDL and LL37-LDL complexes in detail, we used a simple model of LDL particles as an ellipsoid with a concentric core of cholesterol esters (51–53) (Supplemental Figure 1, B and C). The best fits and the model parameters describing the overall size and shape of LDL particles and LDL complexes are summarized in Supplemental Figure 1, B and C. LDL particles have overall dimensions of ~220.4 Å × 95 Å, which is equivalent to a sphere with a diameter of d_sphere_~264 Å, while maintaining the same surface area as the ellipsoid (Supplemental Figure 1C). This result is in rough agreement with the previously reported LDL dimensions using cryogenic transmission electron microscopy (cryo-TEM) (50–53). As expected from the SAXS data, upon the interaction of LDL particles with mouse Cramp, the size of the LDL particles only slightly increased to ~243 Å × 100.8 Å, corresponding to d_sphere_ ~286 Å (Figure 2G). However, the interaction between LDL particles and LL34 and LL37 led to a substantial increase in the LDL size to ~298.2 Å × 106.5 Å (d_sphere_~323 Å) and ~305 Å × 110 Å (d_sphere_ ~332 Å), respectively (Figure 2G). This enlargement of LDL particles by LL37, but not by Cramp, would provide a larger surface area for LDL to bind to the cell surface and also reduces the membrane-bending energy in receptor-mediated endocytosis (50, 54). Thus, these observations were consistent with the greater capacity of LL37 to enhance LDL binding when compared with Cramp.

To confirm the binding of LL37 to LDL, a mixture of biotinylated LDL and LL37 was subjected to coimmunoprecipitation and immunoblotting. Immunoblotting for LL37 after pulldown of LDL showed that LL37 was coprecipitated by LDL, confirming the LL37-LDL interaction (Figure 2H). However, when the mixture of biotinylated LDL with Cramp was subjected to pulldown of LDL, subsequent immunoblotting did not detect the presence of Cramp in the precipitate (Figure 2I). These results confirmed that LL37, but not Cramp, binds to LDL.

To better understand how the peptide charge and hydrophobicity of LL37 affected LDL uptake, we next compared the capacity of single amino acid substitutions in LL34 to alter LDL cell entry. Analysis of LDL uptake into THP-1 cells after addition of an alanine scan mutant library of LL34 peptides showed that substitutions at F5A, F6A, K10A, and I13A resulted in a greater than 50% reduction of their capacity to increase LDL uptake, and substitutions at K25A, F27A, and L28A had between a 30% and 50% reduction of their capacity to increase LDL uptake (Figure 2J). Mapping each of the amino acid positions that affected LDL entry on a helical wheel plot (circled in green in Figure 2K) revealed that alanine substitutions located on the hydrophobic face of the predicted α-helical structure of LL37 had the most influence on LDL uptake, and some but not all substitutions of cationic amino acids also decreased activity (Figure 2K). These structure-function studies suggest that the hydrophobic face and charge position within LL37 are both important (Figure 2, J and K).

Immunofluorescence microscopy of the mixture of Dil-LDL with LL37 in a cell-free buffer showed that LL37 could form visible LDL aggregates over time (Figure 2L). We therefore wished to compare whether the aggregate formation correlated with LDL uptake activity. Several LL34 mutant peptides showed more than a 50% reduction of LDL aggregates compared with the parent peptide (Figure 2L and Supplemental Figure 1D). Ten of the 15 mutant peptides that showed more than a 50% reduction of LDL aggregates had an amino acid substitution in hydrophobic amino acids (Figure 2, L and M, and Supplemental Figure 1D). However, LDL uptake (Figure 2J) did not correlate well with the capacity to promote visible aggregate formation (Figure 2N and Supplemental Figure 1D). Furthermore, phosphatidylcholine (PC) blocked LL37-induced LDL aggregate formation (Supplemental Figure 1, E and F) but did not block LL37-induced LDL uptake or binding of LL37 to LDL (Supplemental Figure 1, G-I). These results suggest that the capacity to form large aggregates of LDL does not predict the capacity for LL37 to induce LDL uptake and further emphasizes the importance of single particle interactions in the uptake process, given the LL37-LDL particle shape changes measured by SAXS.

### LDL uptake after LL37 requires endocytosis and association with cell-surface LDL receptors.

**Figure 3:**
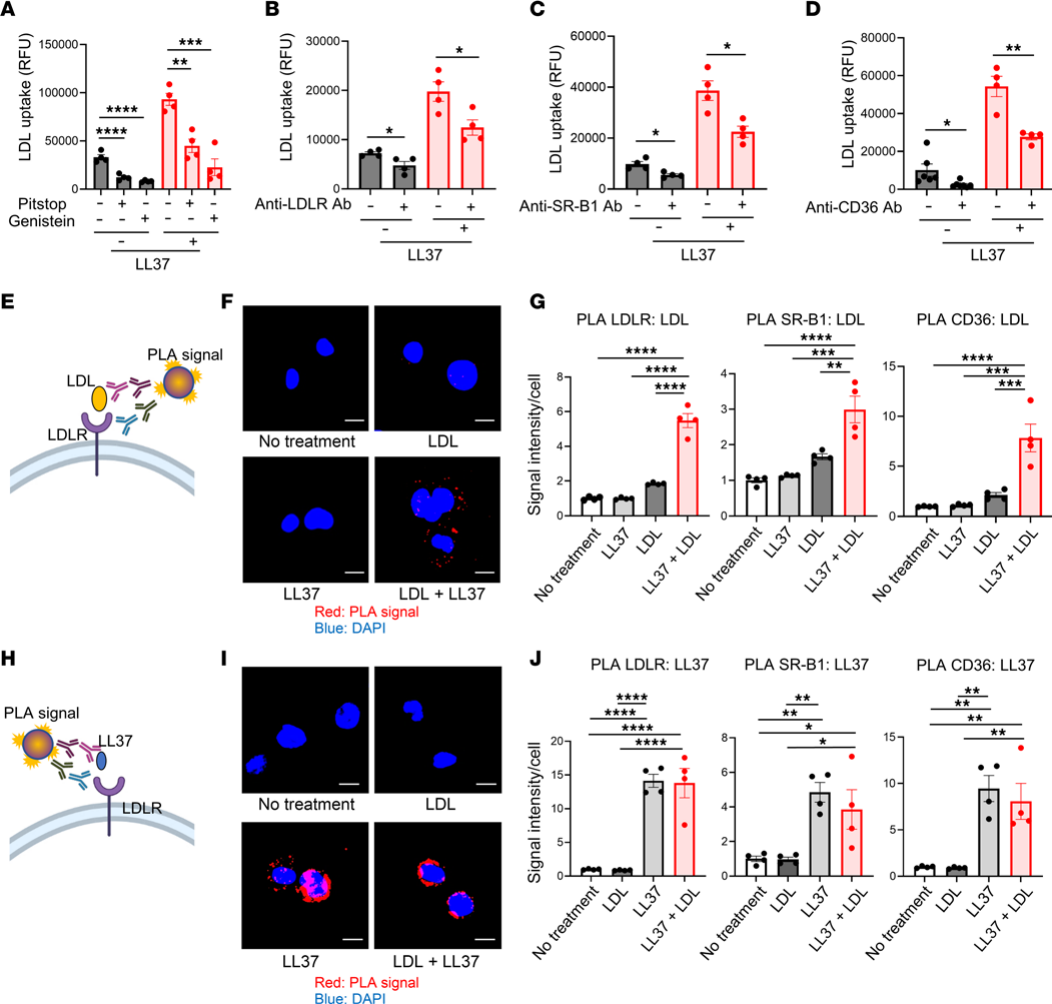
LL37 enhances the binding of LDL to its receptors. (**A**–**D**) pHrodo-LDL uptake into THP-1 cells ± LL37 after pretreatment with Pitstop or Genistein (**A**), anti-LDLR antibody (**B**), anti–SR-B1 antibody (**C**), or anti-CD36 antibody (**D**) (*n* = 4–7 per group). (**E**–**G**) PLA between LDL and LDL receptors of THP-1 cells treated with biotinylated LDL with or without LL37. Schema (**E**), representative PLA images detecting an association between LDL and LDLR (**F**), and fluorescence quantification of positive signal (*n* = 4 per group) (**G**). (**H**–**J**) PLA between LL37 and LDL receptors of THP-1 cells treated with LDL with or without LL37. Schema (**H**), representative images detecting association between LL37 and LDLR (**I**), and fluorescence quantification of positive signals (*n* = 4 per group) (**J**). Scale bars: 10 μm. Data indicate the mean ± SEM. *P < 0.05, **P < 0.01, ***P < 0.001, and ****P < 0.001, by Dunnett’s test (**A**), 2-tailed Student’s *t* test (**B**–**D**), or 1-way ANOVA multiple-comparison test (**G** and **J**).

To understand how LL37 promotes LDL uptake into the cytosol and to determine whether LL37 increases binding of LDL to the cell surface, we next tested the effects of endocytosis inhibitors and blocking antibodies against the LDL receptor (LDLR), scavenger receptor class B member 1 (SR-B1), and CD36, known cell-surface receptors responsible for LDL uptake (36). The endocytosis inhibitors Pitstop and Genistein each strongly suppressed LL37-induced LDL uptake into THP-1 cells (Figure 3A). Furthermore, receptor-blocking antibodies for LDLR, SR-B1, and CD36 each also suppressed LL37-induced LDL uptake into THP-1 cells (Figure 3, B–D). We also observed significant suppression of LL37-induced LDL uptake by these receptor-blocking antibodies in HMDMs (Supplemental Figure 2, A–C). These results suggest that LL37-induced LDL uptake required endocytosis and was mediated in part by the known LDL receptors LDLR, SR-B1, and CD36 in the macrophages.

Next, to further establish the capacity of LL37 to facilitate binding of LDL to its receptors, we assessed the localization of LDL to LDLR, SR-B1, and CD36 by proximity ligation assay (PLA) (spatial correlation <40 nm). We found that LL37 increased the magnitude of a positive PLA signal for LDL with each of its receptors in both THP-1 cells and HMDMs (Figure 3, E–G, and Supplemental Figure 2D). However, LL37 associated with each of the LDL receptors, even without the addition of LDL (Figure 3, H–J, and Supplemental Figure 2E). In contrast to LL37, mouse Cramp did not promote close localization of LDL with LDL receptors, although, like LL37, Cramp associated with the LDL receptors in THP-1 cells (Supplemental Figure 2, F and G). LDL binding activity to the cell surface was also tested at 4°C to slow receptor internalization, and LL37, but not Cramp, increased LDL binding to the cell surface in both THP-1 cells and HMDMs (Supplemental Figure 2, H and I). These results show that, although LL37 and Cramp could each associate with the cell-surface receptors, only LL37 enhanced binding of LDL to its receptors. This observation was again consistent with the unique LL37-LDL particle shape changes measured by SAXS and observations that receptor-mediated endocytosis was required for LL37 to increase LDL internalization.

### LL37 increases cholesterol uptake and alters the transcriptional response to LDL.

**Figure 4:**
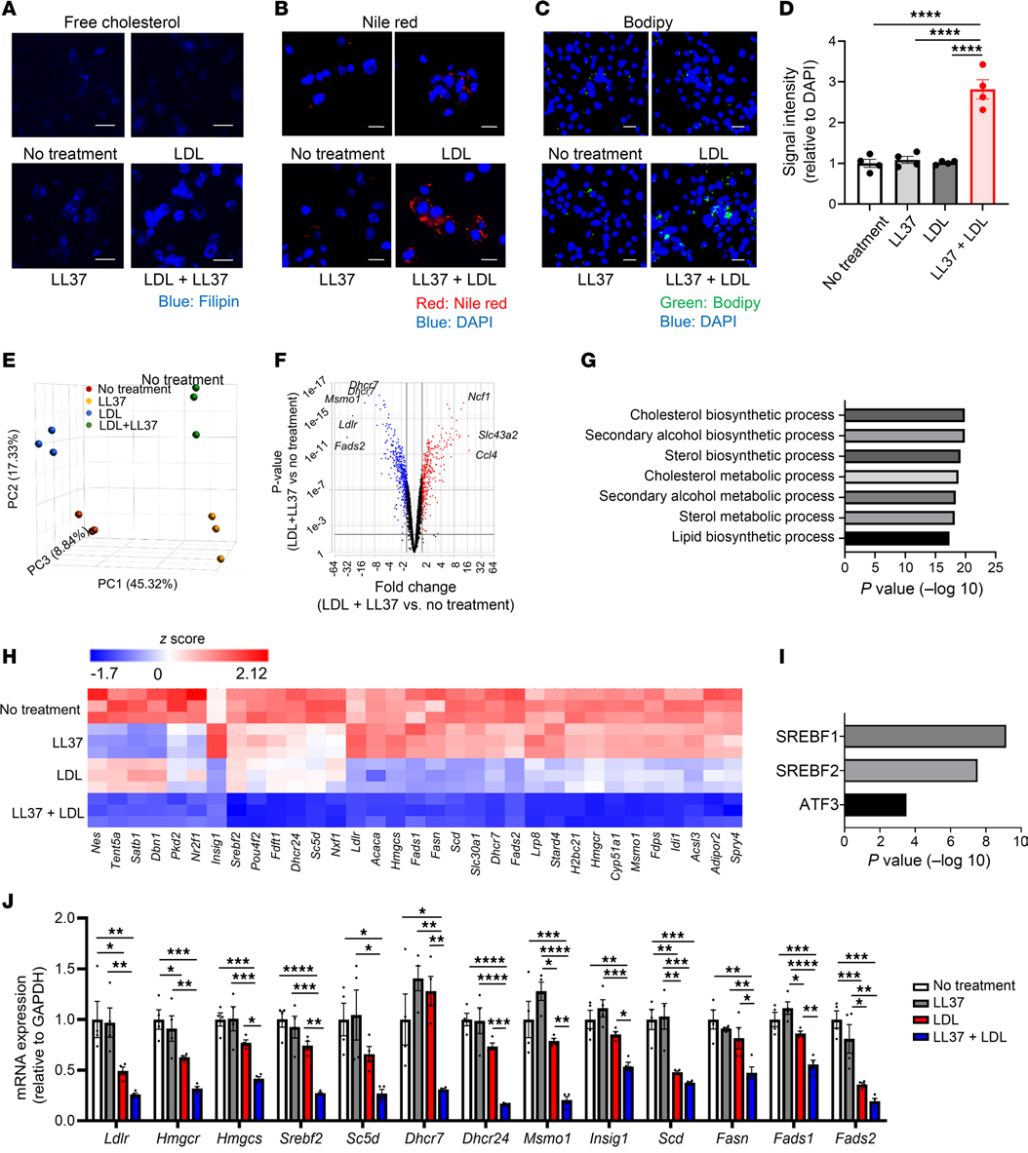
LL37 and LDL increase intracellular lipid and alter macrophage gene expression. (**A**–**C**) Representative images of THP-1 cells treated with LDL with or without LL37 after staining with filipin (blue) to detect free cholesterol (**A**), or with Nile red (red) to detect lipids, and with DAPI (blue) to detect DNA (**B**), or with Bodipy (green) to detect lipids and DAPI (blue) to detect DNA (**C**). Scale bars: 50 μm (**A**) and 20 μm (**B** and **C**). (**D**) Quantitative analysis of signal intensity in THP-1 cells after Bodipy staining as in **C** (*n* = 4 per group). (**E**–**I**) RNA-Seq analyses of THP-1 cells treated with LDL with or without LL37 for 24 hours (*n* = 3 per group). (**E**) PCA plot of the transcriptional profile. (**F**) Volcano plot of differentially expressed genes between no treatment and LDL plus LL37. (**G**) GO term analysis and (**H**) heatmap visualization of selected genes downregulated by LDL plus LL37 treatment compared with LDL or LL37 monotherapy. (**I**) Transcription factors predicted to influence the expression of genes shown in **H**. (**J**) qPCR quantification of mRNA expression for the indicated genes in THP-1 cells treated with LDL with or without LL37 (*n* = 4 per group). Data indicate the mean ± SEM. *P < 0.05, **P < 0.01, ***P < 0.001, and ****P < 0.001, by 1-way ANOVA multiple-comparison test.

Compared with treatment with LDL alone, staining for unesterified cholesterol increased in cells treated with LDL and LL37 in both THP-1 cells and HMDMs (Figure 4A and Supplemental Figure 3A). Furthermore, we observed strong Nile red and Bodipy staining for lipid accumulation under these conditions, suggesting that early foam cell formation could occur in cells treated with LDL plus LL37 compared with the other 3 groups (Figure 4, B–D, and Supplemental Figure 3, B–D).

Since increased uptake of cholesterol into cells is known to result in changes in gene expression that include feedback suppression of lipid synthesis (55), we next assessed global transcriptomic changes in THP-1 cells 24 hours after addition of LDL and LL37. Principal component analysis (PCA) of bulk RNA-Seq results revealed that cells treated with LDL plus LL37 had a substantially different gene expression profile than after addition of either LDL or LL37 alone (Figure 4E). Volcano plots of differentially expressed genes showed that LDL plus LL37 treatment resulted in the downregulation of *Ldlr*, *Fads2*, *Msmo1*, and *Dhcr7* genes associated with the metabolism of cholesterol or fatty acids (Figure 4F). In 33 genes identified by RNA-Seq to be downregulated by LDL plus LL37 treatment compared with LDL or LL37 monotherapy, gene ontology (GO) term analysis showed that the top 7 downregulated gene annotation sets were metabolic or biosynthetic processes consistent with the cellular response to increased intracellular cholesterol (Figure 4, G and H). SREBF1 and SREBF2, master regulators to promote the synthesis of cholesterol and fatty acids (56), were predicted to be transcription factors that control these gene sets (Figure 4I). The selected genes associated with lipid metabolism (*Ldlr*, *Hmgcr*, *Hmgcs*, *Srebf2*, *Sc5d*, *Dhcr7*, *Dhcr24*, *Msmo1*, *Insig1*, *Scd*, *Fasn*, *Fads1*, and *Fads2*) were confirmed by quantitative PCR (qPCR) to be decreased by LL37 plus LDL treatment (Figure 4J). All of the selected genes except for *Sc5d* were also downregulated by LL37 plus LDL treatment in HMDMs (Supplemental Figure 3E). These results support the observations that LL37 increased LDL-derived cholesterol in the cytosol and the subsequent transcriptional response by macrophages.

### LL37 enhances the development of atherosclerosis in mice.

**Figure 5:**
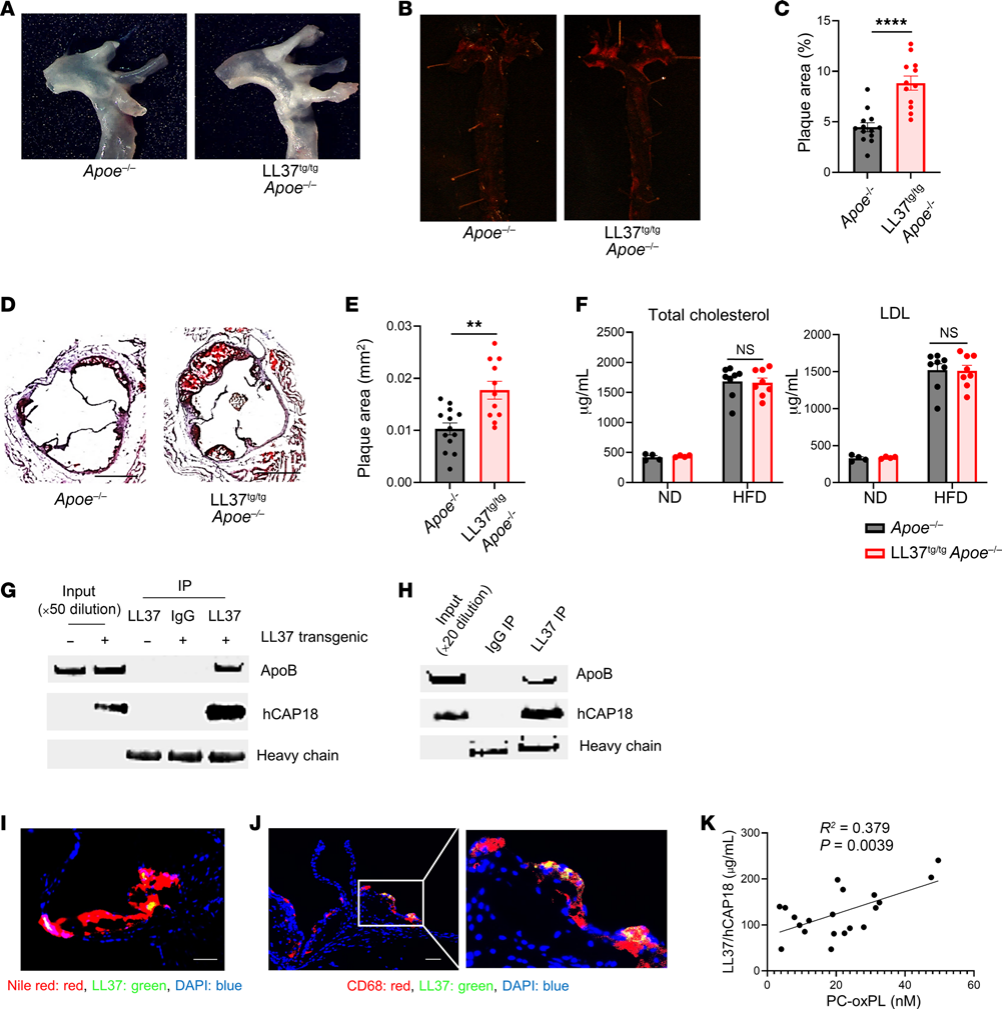
Transgenic expression of *CAMP* enhances the development of atherosclerosis. (**A**–**F**) *Apoe^–/–^* and LL37^tg/tg^ *Apoe^–/–^* mice were fed a HFD for 10 weeks. (**A**) Representative images of the aortic arch. (**B** and **C**) Representative en face images of aortas stained with oil red (**B**) to detect atherosclerotic plaques and quantitation of lesion surface area (**C**) (*n* = 13 in *Apoe^–/–^* mice, *n* = 12 in LL37^tg/tg^ *Apoe^–/–^* mice). (**D** and **E**) Representative images of oil red/hematoxylin-stained aortic sinus sections (**D**) and quantitation of plaque area (**E**) (*n* = 13 in *Apoe^–/–^* mice, *n* = 11 in LL37^tg/tg^ *Apoe^–/–^* mice). Scale bar: 500 μm (**D**). (**F**) Mouse serum concentrations of total cholesterol and LDL cholesterol (*n* = 4 per group fed a ND, *n* = 8 per group fed a HFD, respectively). (**G**) Coimmunoprecipitation of serum from *Apoe^–/–^* mice or LL37^tg/tg^ *Apoe^–/–^* mice fed a ND with anti-LL37 and detection with anti-LL37 and anti-apoB. (**H**) Coimmunoprecipitation of human serum from healthy blood donors with anti-LL37 and detection with anti-LL37 and anti-apoB. (**I** and **J**) Representative images of Nile red/LL37–stained plaques (**I**) and CD68/ LL37-stained plaques (**J**) in LL37^tg/tg^ *Apoe*^–/–^ mice. Scale bars: 50 μm (**I** and **J**). Original magnification, ×3 (enlarged inset on right in **J**). (**K**) Linear regression analysis of human plasma LL37 and PC-oxPL in patients with atherosclerosis (*n* = 20). Data indicate the mean ± SEM. **P < 0.01 and ****P < 0.0001, by 2 tailed Student’s *t* test (**C** and **E**) or linear regression analysis (**K**).

To examine whether LL37 could promote the development of atherosclerosis, we next crossed LL37^tg/tg^ mice with *Apoe^–/–^* mice and assessed the development of atherosclerotic plaques in mice after 10 weeks of a high-fat diet (HFD). Plaques were visualized by in situ images of the aortic arch and lipid staining en face of the thoracic aorta. LL37^tg/tg^*Apoe^–/–^* mice showed an increase in plaque size in the aorta compared with control *Apoe^–/–^* mice that lacked *CAMP* (Figure 5, A–C). Lipid-stained sections of the aortic sinus revealed a larger plaque size in LL37^tg/tg^ *Apoe^–/–^* mice compared with controls (Figure 5, D and E). Body weight changes during feeding of a normal diet (ND) and a HFD were similar between LL37^tg/tg^ *Apoe^–/–^* and control *Apoe^–/–^* mice (Supplemental Figure 4A). Circulating total cholesterol, LDL, HDL, and triglycerides were also comparable between the groups (Figure 5F and Supplemental Figure 4B). Previous studies have shown that the cathelicidin precursor protein hCAP18 can bind to lipoproteins, including VLDL, LDL, and HDL, in human serum through the LL37 domain at the C-terminus before LL37 is cleaved from hCAP18 (30–32, 57). To assess whether LL37/ hCAP18 in the serum of LL37^tg/tg^ *Apoe^–/–^* mice could bind to LDL, mouse serum was subjected to coimmunoprecipitation and immunoblotting. Immunoblotting for apolipoprotein B (apoB) after pulldown of LL37 showed that apoB was coprecipitated by LL37 (Figure 5G). Similarly, immunoblotting with anti-LL37/hCAP18 antibody detected hCAP18 (Figure 5G). As an alternative approach to establishing the association of LL37 with lipoprotein particles in these transgenic mice, the serum of LL37^tg/tg^ *Apoe^–/–^* mice was size separated by fast protein liquid chromatography (FPLC) from other serum components. Analysis of the lipoprotein distribution fractions showed that LL37/hCAP18 was present mainly in fractions of apoB-containing lipoproteins including VLDL/chylomicron, intermediate-density lipoprotein (IDL), and LDL, although we also detected smaller amounts of LL37/hCAP18 in HDL fractions (Supplemental Figure 4, C–E). Immunoblotting of human serum from a healthy donor for apoB after pulldown of LL37 also demonstrated that apoB was coprecipitated by LL37 (Figure 5H). These results establish that LL37/ hCAP18 bound to apoB-containing lipoproteins including the atherogenic IDL and LDL particles in both human serum and serum from LL37^tg/tg^ *Apoe* *^–/–^* mice.

LL37^tg/tg^ *Apoe^–/–^* mice showed accumulation of LL37 in the atheroma plaque, and LL37 was mainly present around macrophages (Figure 5, I and J, and Supplemental Figure 4F). To explore whether the observations of an increased risk for plaque formation seen in mice may also correlate with cardiac risk in human samples, we measured fresh human plasma LL37/ hCAP18 levels in patients with atherosclerosis. The concentration of LL37/hCAP18 positively correlated with PC–oxidized phospholipid (PC-OxPL) levels, a predictive factor for the development and progression of atherosclerosis (58) (Figure 5K). Overall, these results support the hypothesis that LL37, which is elevated in patients with some inflammatory disorders such as psoriasis, rosacea, IBD, and RA, contributes to the increased risk of atherosclerosis in these patients.

## Discussion

In this study, we show that LL37 could promote LDL uptake into cells and tissues, and suggest a mechanism for this process by demonstrating that LL37 bound to LDL to form a structure different from that of peptides, which do not promote LDL uptake. LL37 remodeled the geometry of LDL to facilitate its uptake through classical LDL receptors such as LDLR, SR-B1, and CD36 and was then actively internalized to drive a greater accumulation of lipids in these cells. We also show that in mice, transgenic expression of LL37 resulted in increased development of atherosclerotic plaques. Given that LL37 levels are increased in chronic inflammatory disorders such as psoriasis, this may explain the increased risk of atherosclerotic disease in patients with inflammatory skin diseases (3–6, 8–11, 59, 60).

The cathelicidin gene family is ancient and is ubiquitously present in diverse species including mammals, chickens, amphibians, and fish (61, 62). Some cathelicidin peptides have dual activities and function as innate antibiotics as well as various immunomodulatory effects such as neutralization of endotoxins and promotion of uptake and TLR-mediated recognition of nucleic acids (63, 64). In addition, cathelicidin peptides can function to activate receptors such as the formyl peptide receptor 2 (FPR2) and P2X7, resulting in chemotactic and proinflammatory properties (20, 65). However, although proteins in the cathelicidin gene family are highly conserved in the precursor domain, evolution has resulted in great diversity in the C-terminal peptide domains so the mature cathelicidin peptides have different functions between species. In general, although cathelicidin peptides maintain antimicrobial function, they show variation in functions related to cell activation and proinflammatory activity. For example, whereas human LL37 induces P2X7 activation, mouse Cramp does not (65). We observed that human cathelicidin peptides from primates most closely related to humans could promote LDL uptake but that cathelicidin peptides from more distantly related species did not. A similar divergence between the capacity to promote inflammation in response to DNA and dsRNA has been previously seen between LL37 and mouse Cramp (66). Furthermore, prior structure-function studies of LL37 have shown that amino acid residues critical for LL37 to promote cytokine release (49) are similar to the residues that are important for LDL uptake. However, there is some discrepancy between mutant peptides that have reduced activity for LDL uptake and activation of cytokine expression in response to nucleic acids. For example, whereas LL34-I24A and L31A have a greatly reduced capacity to induce expression of *Il6*, *Ifnb1*, and *Cxll10* (49), such mutant peptides had comparable or higher activity for LDL uptake. Thus, the sequence determinants that dictate the inflammatory activity of cathelicidin peptides are not identical to those that promote LDL uptake.

An interesting and unanticipated function of LL37 reported here is its ability to enforce an increase in the effective size of LDL, given that the density of circulating lipoproteins is related to their functional classifications (ex: VLDL, IDL, LDL, HDL). LL37 is a canonical antimicrobial peptide, a class of innate immune molecules known to permeate membranes by generating negative Gaussian curvature in membranes (67, 68). In this context, it is interesting to note an increase in the size of LDL amounts to a reduction of positive Gaussian curvature on the lipoprotein surface, which can be related to the negative Gaussian curvature generation capacity of LL37. More generally, that LL37 can increase the size of LDL via curvature remodeling and thereby affect LDL uptake suggests that there may be other connections between innate immunity and functions of lipoproteins. We are currently working to formalize these concepts.

Several mechanisms have been proposed to explain the mechanism for how LL37 promotes uptake of nucleic acids (69, 70), but these may not apply to the process of LDL uptake. One of the suggested mechanisms is that LL37 interacts and stabilizes nucleic acids, resulting in protection of the nucleic acids from degradation by enzymes such as DNases and RNases (69, 70). However, this model is unlikely for LDL uptake. Also, as discussed earlier, comparison of results with LL34 mutants showed some distinctions between amino acid residues that induce dsRNA and lipoprotein uptake. Another model has suggested that ordering of nucleic acids in LL37 complexes promotes multivalent binding with cell-surface receptors such as scavenger receptors (49). This model needs to be explored more completely for LDL-LL37 complexes. Finally, the exposed cationic residues of LL37 (69, 70) may enable attachment of LDL to the cell surface. This model is less likely for LDL, as we observed that Cramp and LL37 associated equally well with LDL surface receptors and did not require the presence of LDL, thus making the role of charge alone an unlikely explanation for increased LDL binding. Ongoing work to explore these models can further define the critical structures required for LDL binding.

Our observations provide insight into prior findings that have shown that LL37 accumulates in atheroma plaques (71). We now show LL37^tg/tg^ mice on the *Apoe^–/–^* background increased the development of atherosclerotic plaques compared with control *Apoe^–/–^* mice that lacked LL37, indicating a specific effect from the human cathelicidin gene product. Although the exact mechanisms for how LL37 may affect the development of atherosclerosis remains unclear, the phenotype of LL37^tg/tg^ mice was not associated with elevations of serum cholesterol and triglyceride, suggesting that this was not a mechanism to explain the formation of atherosclerotic plaques. Given the specific effect of LL37 in promoting LDL uptake into cells, which is not observed with mouse cathelicidin, we propose that the presence of LL37 in LL37^tg/tg^ mice likely resulted in macrophage-driven uptake of the LL37-LDL complex and the observed increase in atherosclerotic plaques. However, there are also other mechanisms for the observation of increased plaques in LL37^tg/tg^ mice. Since LDL aggregation contributes to the progression of atherosclerosis via increased LDL retention and overall plaque burden (72), it is also possible that atherosclerosis may have been driven by LDL aggregation. It is also important to note that it has previously been reported that a lack of mouse cathelicidin in *Camp^–/–^* mice can reduce the development of atherosclerosis in mice (73). As we have shown that mouse cathelicidin did not directly increase LDL uptake, it is possible that these observations were a consequence of the effects of mouse cathelicidin in increasing inflammation (74) and that the elimination of mouse cathelicidin improved disease because of lesser inflammation. In our model of LL37^tg/tg^ mice on the *Apoe^–/–^* background, the enhanced atheroma formation may therefore have been due to proinflammatory activities of LL37 such as its increased capacity to promote P2X7 activation compared with mouse cathelicidin (65). Thus, while our observations showed that expression of a human-specific AMP in mice could promote the development of atherosclerosis, it may influence this event through multiple mechanisms including effects on inflammation, LDL aggregation, or LDL uptake.

Some prior studies have suggested a potential protective role of LL37 against atherosclerosis-induced cardiovascular events (75–78). Bei et al. showed that serum levels of LL37/ hCAP18 are lower in patients with myocardial infarction than the levels in healthy individuals (77). A prospective study conducted by Zhao et al. reported that high basal plasma levels of LL37/ hCAP18 predict a lower risk of atherosclerosis-induced cardiovascular events in patients after ST-elevation myocardial infarction (75). However, these observations were made in acute settings that may have reflected the beneficial roles of cathelicidin during tissue repair and host defense, not the chronic risk of prolonged elevation of LL37. Our chronic expression model aligns well with prior reports demonstrating that plasma concentrations of LL37/ hCAP18 are significantly higher in patients with atherosclerosis compared with those in healthy volunteers (74). This is also consistent with our observation of a positive correlation between plasma LL37/ hCAP18 and PC-oxPL levels, a potent predictive factor for the development and progression of atherosclerosis (58).

Our study has some limitations that should be considered. Although we propose that LL37-induced LDL uptake is one of the mechanisms for the increased plaque size in LL37^tg/tg^ mice on the *Apoe^–/–^* background compared with control mice, there is also a possibility of involvement of other mechanisms such as LL37-induced LDL aggregates or LL37-induced inflammation as described earlier. In addition, although we showed that the presence of LL37 promoted the development of atherosclerosis in mice, the role of LL37 in the development of human atherosclerotic plaque has yet to be determined, since several aspects of the pathogenesis of atherosclerosis differ between humans and mice (79, 80). Furthermore, although a positive correlation between plasma LL37/ hCAP18 and PC-oxPL levels was observed in patients with atherosclerosis, whether the correlation will also be observed in the plasma of patients with chronic inflammatory disorders such as psoriasis remains unclear. Despite these limitations, our study describes a new potential mechanism by which LL37 can participate in the development of atherosclerosis.

In conclusion, this study shows that LL37, an AMP specific to humans, has the capacity to promote LDL uptake into cells and can increase the development of atherosclerosis in mice. These observations may explain why patients with chronic inflammatory disorders such as psoriasis, rosacea, IBD and RA that produce large amounts of LL37 have a greater risk of cardiovascular diseases. Future studies may uncover diagnostic or therapeutic applications for the targeting of LL37 in atherosclerosis.

## Methods

Additional details on methods can be found in the Supplemental Methods.

### Mice.

C57BL/6 WT mice and *Apoe*-KO mice (*Apoe^–/–^*) were obtained from The Jackson Laboratory. Cathelicidin-KO mice (*Camp^–/–^*) were generated in our laboratory as previously described (81). Human cathelicidin-transgenic mice (48, 82) were bred with mice on a *Camp^–/–^* background to generate LL37^tg/tg^ and LL37^tg/tg^ *Apoe^–/–^* mice for use in the animal studies. Mice between 6 and 10 weeks of age were used for the LDL uptake experiments for LDL. In studies of atherosclerosis, male mice at 6 age weeks received an atherogenic diet (Teklad TD 88137, 21% fat, 0.2% cholesterol; Harlan Laboratories) for 10 weeks.

### Cell culturing.

THP-1 cells, EA.hy926 cells, HUVECs, and HAoECs were obtained from the American Type Culture Collection (ATCC). THP-1 cells were maintained in RPMI-1640 (Thermo Fisher Scientific) supplemented with 10% FBS and antibiotic-antimycotic (Thermo Fisher Scientific). THP-1 cells at 60%–80% confluence were differentiated by PMA (Abcam) for 24 hours and then starved overnight without FBS prior to treatment. EA.hy926 cells were maintained in DMEM (Thermo Fisher Scientific) supplemented with 10% FBS and antibiotic-antimycotic, and the cells were seeded in Endothelial Cell Growth Medium MV2 (PromoCell) 24 hours before treatment. HUVECs were maintained in Endothelial Cell Growth Medium (PromoCell) supplemented with antibiotic-antimycotic. HAoECs were maintained in Endothelial Cell Growth Medium MV supplemented with antibiotic-antimycotic. To generate HMDMs, human primary monocytes were isolated from peripheral blood mononuclear cell obtained from healthy blood donors using density gradients. CD14^+^ cells purified with MACS bead (Miltenyi Biotec) were cultured in RPMI-1640 supplemented with 10% FBS, antibiotic-antimycotic and 40 ng/mL M-CSF (Thermo Fisher Scientific) for 7 days for differentiation into macrophages. After the culture, experiments were conducted with serum free RPMI-1640 with antibiotic-antimycotic and 40 ng/mL M-CSF.

### Chemicals and reagents.

Phosphatidylcholine (PC) was purchased from Sigma Aldrich, and used for pretreatment with 20 min incubation at a concentration of 2 μg/mL. DMSO was used as a vehicle. Dil-LDL and native LDL were purchased from Thermo Fisher Scientific. Native VLDL and native HDL were purchased from Kalen Biomedical and Abcam, respectively. Rabbit anti-Cramp and rabbit anti–LL-37 antibodies were made in our laboratory as previously described (83). Synthetic LL-37 and Cramp were purchased from Genemed Synthesis. Synthetic LL34 Alanine Scan Peptides and cathelicidin peptides of gorilla, gibbon, rhesus monkey, marmoset (Callithrix jacchus), and rabbit were purchased from LifeTein. The sequences of the cathelicidin peptides used in this study are shown in Supplemental Table 1. Recombinant Human IL-26 was purchased from R&D Systems. Cathelicidin peptides, LL34 alanine scan peptides, and IL-26 were used at a concentration of 5 μM unless otherwise specified.

### Biotinylation of LDL.

The buffer of native LDL (Thermo Fisher Scientific) was replaced by PBS using Zeba Micro Spin Desalting Columns, 7K MWCO (Thermo Fisher Scientific) according to the manufacturer’s instructions and was then was incubated with 6.25 mM EZ-Link Sulfo-NHS-Biotin (Thermo Fisher Scientific) for 30 minutes at room temperature. Excessive biotin was also removed using Zeba Micro Spin Desalting Columns, 7K MWCO.

### pHrodo labeling of lipoproteins.

The buffer of native oxLDL, VLDL, and HDL was replaced by 0.1 M sodium bicarbonate, pH 8.4, using Zeba Micro Spin Desalting Columns, 7K MWCO, and was then incubated with 125 μM Molecular Probes pHrodo Red, succinimidyl ester (Thermo Fisher Scientific) for 60 minutes at room temperature. Removal of excessive pHrodo and replacement of the buffer with PBS were conducted using Zeba Micro Spin Desalting Columns, 7K MWCO.

### Human plasma samples and the OxPL-apoB assay.

Twenty random, anonymized human blood samples from individuals with preexisting cardiovascular disease who had previously elevated oxidized phospholipid on apolipoprotein B (OxPL-apoB) values (range, 3.6–49.6 nmol/L, mean [SD], 21.0 [13], 75th percentile <5.0 nmol/L) were used to measure LL37 plasma levels. LL37 plasma levels were measured using an LL37 ELISA kit (Hycult Biotech). Plasma OxPL-apoB levels were measured with an established ELISA as previously described (84).

### Statistics.

Data presented in this study are from 1 representative experiment of at least 2 independent experiments except for data using human blood samples from individuals with preexisting cardiovascular disease (Figure 5K). Statistical significance was determined using a 2-tailed Student’s *t* test, Dunnett’s test, or 1-way ANOVA multiple-comparison test. To examine associations, linear regression analysis was used. Throughout the analysis, probability values of less than 0.05 were considered significant. The statistical tests were carried out using GraphPad Prism (GraphPad Software). Data in the figures indicate the mean ± SEM.

### Study approval.

All mouse procedures were approved by the UCSD Institutional Animal Care and Use Program (protocol no. S09074). The human study was approved by the UCSD Human Subjects Protection Program.

### Data availability.

All data associated with this study are present in this report or in the supplementary materials, and values for all data points in graphs can be found in the Supplemental Supporting Data Values file. The RNA-Seq data are available in the NCBI’s Gene Expression Omnibus (GEO) database (GEO GSE230360). Materials will be made available upon request.

## Author contributions

Conceptualization: YN, NNK, TT, and RLG. Methodology: YN, NNK, TT, TD, TN, AFG, XY, ST, PS, PLSMG, GCLW, and RLG. Investigation: YN, NNK, TT, TD, EL, MS, EWCL, and HA. Funding acquisition: RLG. Project administration: GCLW and RLG. Supervision: GCLW and RLG. Writing of the original draft of the manuscript: YN and RLG. Editing of the manuscript: NNK, TT, TD, EL, MS, TN, EWCL, HA, AFG, XY, ST, PLSMG, and GCLW.

## Supplementary Material

Supplemental data

Supporting data values

## Version 1 01/09/2024

In-Press Preview

## Version 2 03/01/2024

Electronic publication
