# Supplementary material for: Increased LL37 in psoriasis and other inflammatory disorders promotes LDL uptake and atherosclerosis
Source: J Clin Invest. 2024 Mar 1;134(5):e172578. doi: 10.1172/JCI172578 (PMC10904043; doi:10.1172/JCI172578)
Supplement: Supplemental data [file jci-134-172578-s155.pdf]

Supplementary Material

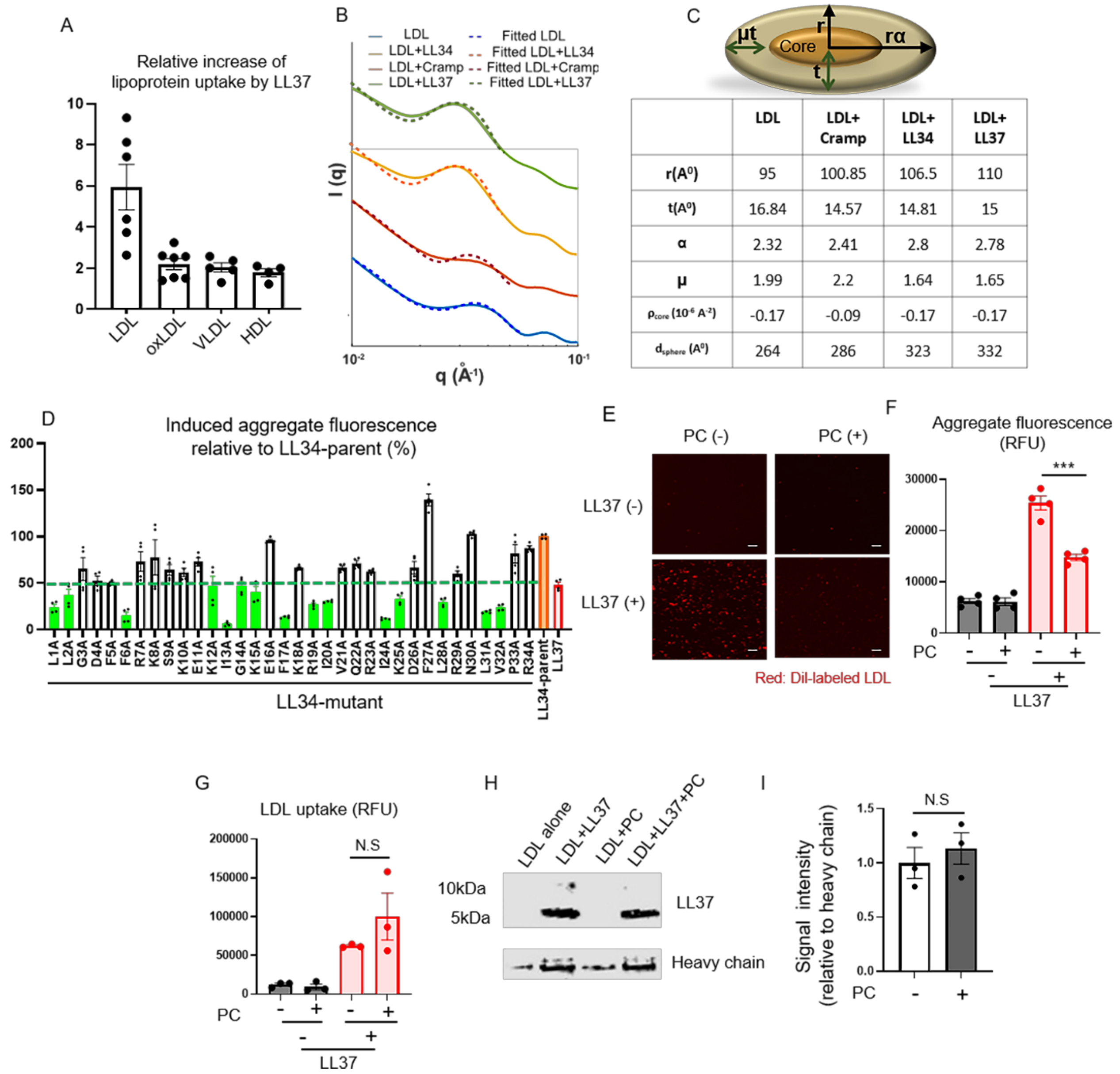

	LDL	LDL+ Cramp	LDL+ LL34	LDL+ LL37
r(A⁰)	95	100.85	106.5	110
t(A⁰)	16.84	14.57	14.81	15
α	2.32	2.41	2.8	2.78
μ	1.99	2.2	1.64	1.65
ρ_{core} (10^{-6} A^{-2})	-0.17	-0.09	-0.17	-0.17
d_{sphere} (A⁰)	264	286	323	332

Supplemental Figure 1. Sequence elements of LL37 that promote uptake of LDL. (**A**) Increase in fluorescence for pHrodo-LDL, pHrodo-oxLDL, pHrodo-VLDL or pHrodo-HDL in THP-1 cells treated with LL37 (n=4-7 per each group). **(B)** Fitted concentric ellipsoidal core-shell model to SAXS profiles of LDL particles and LDL complexes with LL37, LL34 and Cramp. **(C)** The schematic of the ellipsoidal core-shell model and the fitting parameters. (**D)** Quantitative fluorescence analysis of LDL aggregates induced by LL37, LL34 or LL34 mutant peptides with alanine substitutions (LL34 L1A-R34A) (n=4 per each group). Green boxes indicate peptides with specific residues where replacement with an alanine result in more than 50% decrease of LDL aggregate compared to parent LL34 peptide. **(E, F)** Dil-LDL was cultured with LL37 in the presence of PC. Representative fluorescence study of LDL aggregate (E) and quantitative

fluorescence analysis (n=4 per each group) (F) are shown. Scale indicates 20 µm (E). **(G)** Quantitative fluorescence analysis of THP-1 cells treated with pHrodo-LDL and LL37 in the presence of PC (n=3 per each group). **(H, I)** Biotinylated-LDL was cultured with LL37 in the presence of PC. The samples were subjected to co-immunoprecipitation with anti-biotin antibody, and the co-immunoprecipitation samples were then immunoblotted with anti-LL37 antibody. Representative immunoblotting image (H) and quantitative analysis of LL37 signal intensity relative to heavy chain (n=3 per each group) (I) are shown. Error bars indicate mean ± SEM; ***p<0.001 using Student's *t* test. N.S: not significant, SAXS: small angle X-ray scattering, PC: phosphatidylcholine

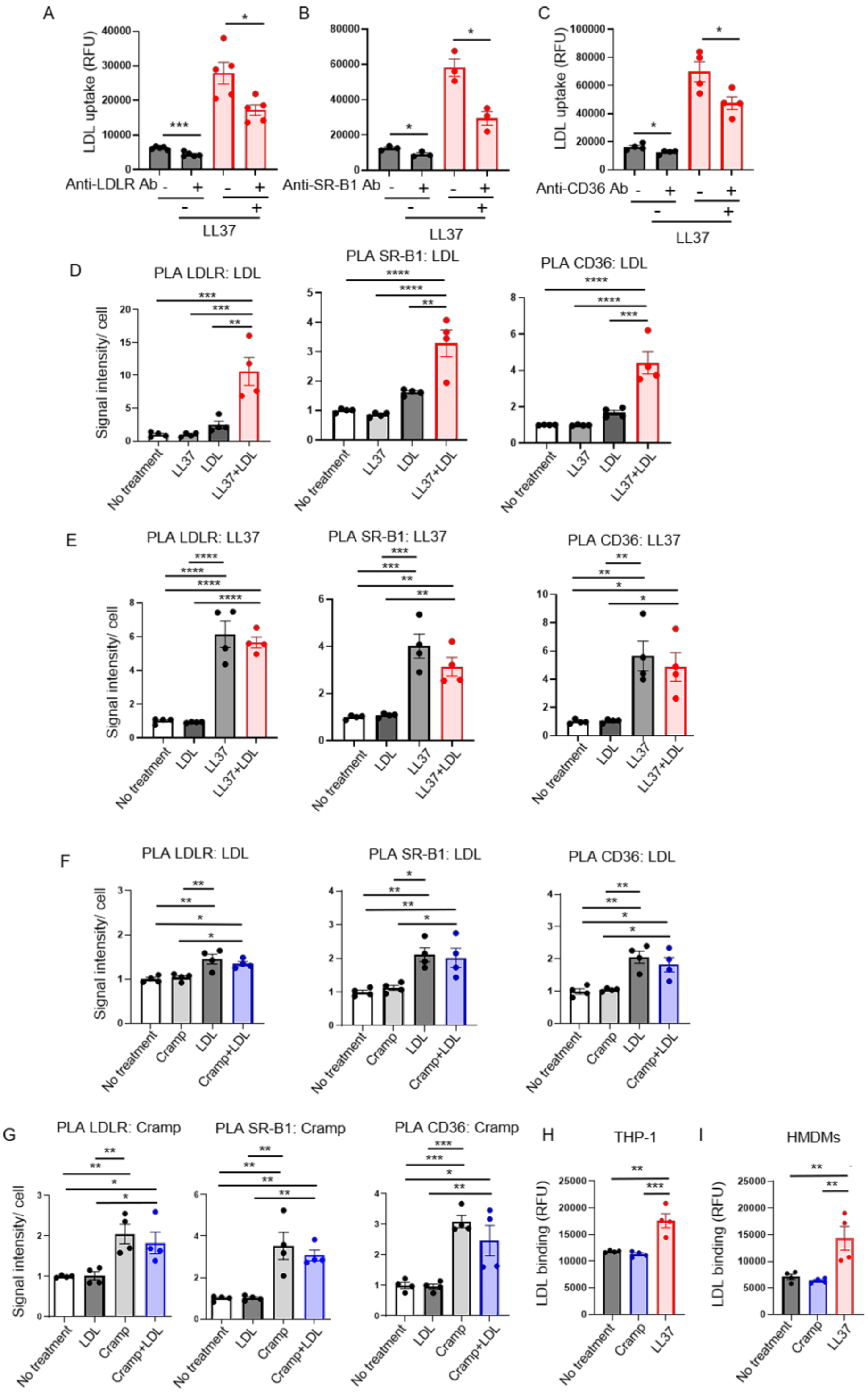

Supplemental Figure 2. LL37 enhances binding of LDL to its receptors.
(A-C) pHrodo-LDL uptake into HMDMs ± LL37 after pretreatment with anti-LDLR antibody (A), anti-SR-B1 antibody (B), or anti-CD36 antibody (C) (n=3-5 per each group). **(D)** Proximity ligation assay (PLA) on HMDMs for biotinylated LDL association with LDLR, SR-B1 or CD36 after addition

of LL37 (n=4 per each group). **(E)** PLA on HMDMs for LL37 associated with LDLR, SR-B1 or CD36 ± LDL (n=4 per each group). **(F)** PLA on THP-1 cells for biotinylated LDL association with LDLR, SR-B1 or CD36 after addition of Cramp (n=4 per each group). **(G)** PLA on THP-1 cells for Cramp associated with LDLR, SR-B1 or CD36 ± LDL (n=4 per each group). **(H, I)** Dil-LDL associated with THP-1 (H) or HMDMs (I) after addition of LL37 or Cramp (n=4 per each group). Error bars indicate mean ± SEM; **p*<0.05, ***p*<0.01, ****p*<0.001, *****p*<0.001 using Student's *t* test (A-C) or one-way ANOVA multiple-comparison test (D-I). HMDMs: human monocyte-derived macrophages

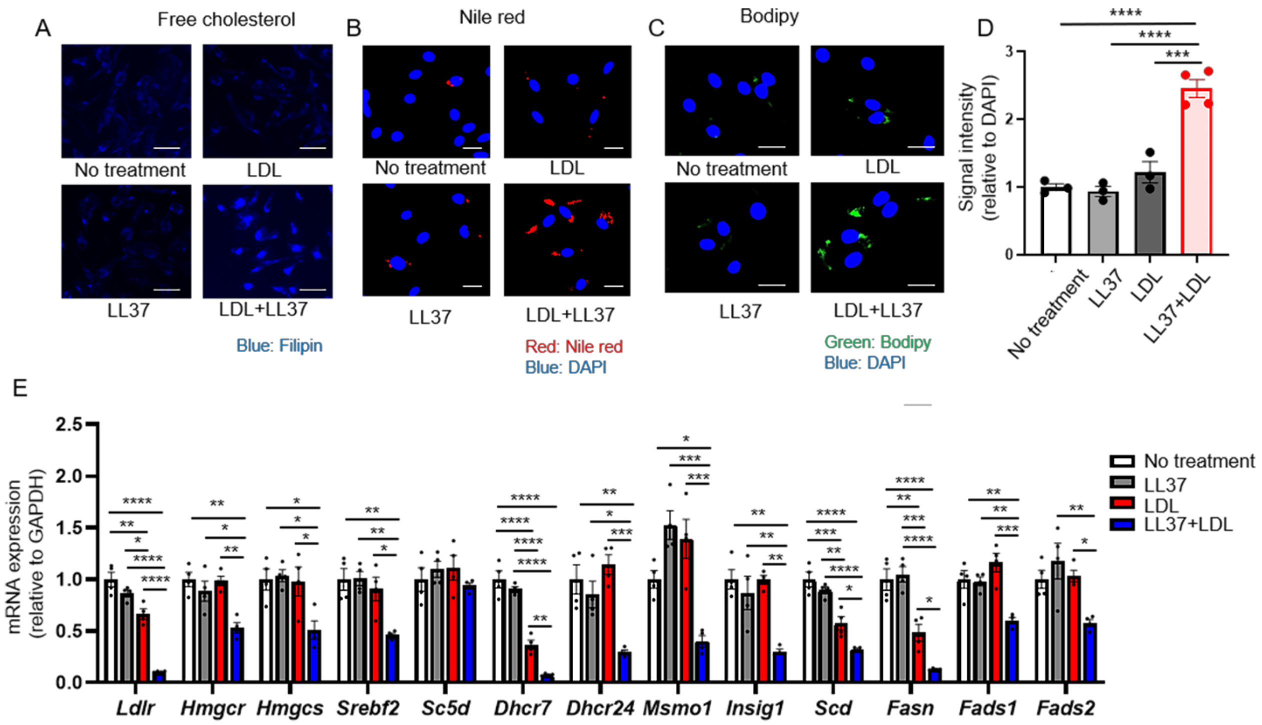

Supplemental Figure 3. LL37 and LDL increases intracellular lipid and alters macrophage gene expression.

(A-C) Representative images of HMDMs treated with LDL ± LL37 after staining with filipin (blue) to detect free cholesterol (A), or with Nile red (red) to detect lipid and with DAPI (blue) to detect DNA (B), or with Bodipy (green) to detect lipids and DAPI (blue) to detect DNA (C). Scale indicates 50 µm (A) or 20 µm (B, C). **(D)** Quantitative analysis of signal intensity in HMDMs after Bodipy staining as in (C) (n=3 or 4 per each group). **(E)** qPCR quantification of mRNA expression for indicated genes in HMDMs treated with LDL ± LL37 (n=4 per each group). Error bars indicate mean ± SEM; **p*<0.05, ***p*<0.01, ***p<0.001, *****p*<0.001 using one-way ANOVA multiple-comparison test. HMDMs: human monocyte-derived macrophages

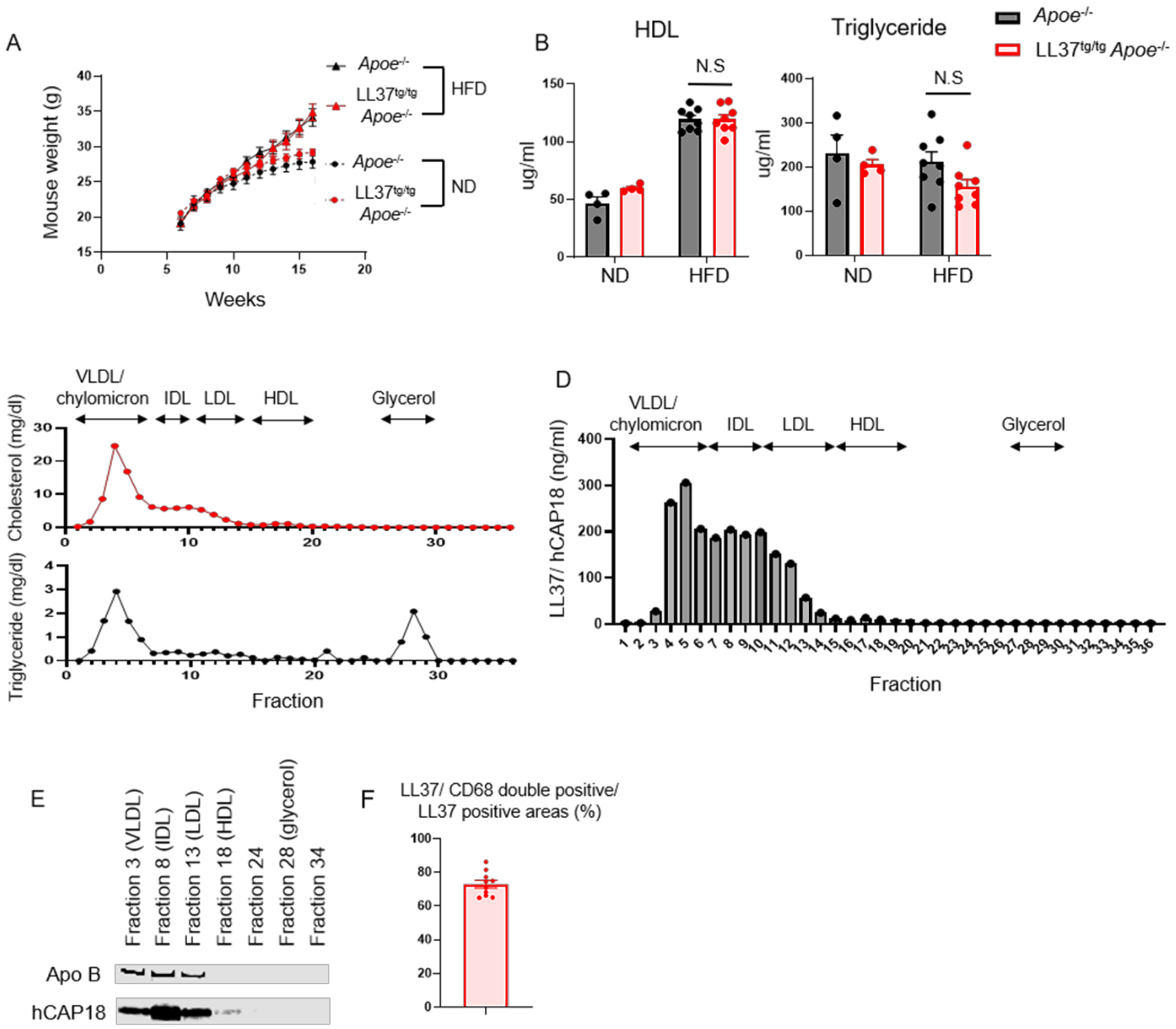

Supplemental Figure 4. Transgenic expression of *CAMP* enhances development of atherosclerosis. (A) Weight change of *Apoe*$^{-/-}$ mice and LL37$^{tg/tg}$ *Apoe*$^{-/-}$ mice fed normal or high fat diet (n=4 and 5 in *Apoe*$^{-/-}$ mice and LL37$^{tg/tg}$ *Apoe*$^{-/-}$ mice with normal diet, respectively. n=8 and 10 in *Apoe*$^{-/-}$ mice and LL37$^{tg/tg}$ *Apoe*$^{-/-}$ mice with high fat diet, respectively). **(B)** Serum concentration of HDL cholesterol and triglyceride in *Apoe*$^{-/-}$ mice and LL37$^{tg/tg}$ *Apoe*$^{-/-}$ mice fed normal or high fat diet for 10 weeks (n=4 per each group with normal diet, n=8 per each group with high fat diet). (**C**) Size-exclusion FPLC (fast protein liquid chromatography) analysis of serum from LL37$^{tg/tg}$ *Apoe*$^{-/-}$ mice with high fat diet to determine cholesterol and triglycerides in each fraction (n=1 per each fraction). (**D**) LL37/ hCAP18 concentration measured by ELISA in each fraction of the FPLC (n=1 per each fraction). (**E**) Immunoblot analyses of indicated fraction of the FPLC with anti-apolipoprotein B (apoB) antibody and anti-LL37 antibody. **(F)** Proportion of LL37/ CD68 stained areas within whole LL37 stained areas in the plaque of LL37$^{tg/tg}$ *Apoe*$^{-/-}$ mice fed high fat diet (n=10). Error bars indicate mean ± SEM; N.S: not significant. ND: normal diet, HFD: high fat diet

Supplemental Table 1. Sequence of cathelicidin peptides

Species	Sequence
Human	LLGDFFRKSKEKIGKEFKRIVQRIKDFLRNLVPRTES
Gorilla	LLGDFFRKAKEKIGKESKRIVQRIKDFLRNLVPRTES
Gibbon	SLGNFFRKARKKIGEEFKRIVQRIKDFLQHLIPRTEA
Rhesus Monkey	RLGNFFRKVKEKIGGGLKKVGQKIKDFLGNLVPRTAS
Common Marmoset	RLGDILQKAREKIEGGLKKLVQKIKDFFGKFAPRTES
Rabbit	GLRKRLRKFRNKIKEKLKKIGQKIQGLLPKLAPRTDY
Mouse	GLLRKGGEKIGEKLKKIGQKIKNFFQKLVPQPEQ

Supplemental Table 2. Sequences and catalog number of PCR primers

Gene	Strand	Sequence	Catalog number
LDLR	Fw	CAGATATCATCAACGAAGC	
	Rv	CCTCTCACACCAGTTCACTCC	
HMGCR	Fw	AGGAGGCATTTGACAGCACT	
	Rv	ACCTGGACTGGAAACGGATA	
HMGCS	Fw	AAGTCCAGGCCAGCAGTGA	
	Rv	ATATTCACAGCTCCTGAATGTACCA	
SC5D	Fw	GGTTGGTTAGCGAGTGCCC	
	Rv	CTGGCCATGTGGCTGGATAC	
MSMO1	Fw	GGCCGTTCAGGATAAGCCAG	
	Rv	CACAACCAAAGCATCTTGCCA	
DHCR24	Fw	TGAAGACAAACCGAGAGGGC	
	Rv	CAGCCAAAGAGGTAGCGGAA	
DHCR7	Fw	ACTTTAGCCGGTTGAGAAGGA	
	Rv	TGGCTTTGGGAATGTTGGGT	
INSIG1	Fw	CATCTTTTCCTCCGCCTGGT	
	Rv	CTGGCGTGGTTAATGCCAAC	
FASN	Fw	ACCTCCGTGCAGTTCTTGAG	
	Rv	GTTCAGGATGGTGGCGTACA	
FADS1	Fw	CGCTACTTCACCTGGGACGAG	
	Rv	TGATGTGGAAGGCCACAAAGG	
FADS2	Fw	TTTGTGTGTGCGTGTTGTTGG	
	Rv	GGGGCCAGTTCACCAATCAG	
GAPDH	Fw	TGGGCTACACTGAGCACCAG	
	Rv	GGGTGTCGCTGTTGAAGTCA	
SREBF2			Hs.PT.58.45335433
SCD			Hs.PT.58.45714389

SUPPLEMENTAL METHODS

Cytochemistry

Cells were treated with LDL (25 μg/ml) and/ or LL37 for 24 hours. Then, the cells were fixed with 4% paraformaldehyde for 10 minutes. For detection of intracellular free cholesterol, the cells were incubated with filipin detection solution (cell-based cholesterol assay kit, Abcam) for 1 hour at room temperature. After that, cells were immediately imaged to prevent photo bleaching. For Nile red staining, fixed cells were stained with Nile red (Thermo Fisher Scientific) for 1 hour at room temperature. For Bodipy staining, fixed cells were incubated with Bodipy 493/ 503 (1: 1000 dilution, Thermo Fisher Scientific) for 30 minutes. Nuclei were counterstained with 4′,6-diamidino-2-phenylindole (DAPI). All images were taken with an Olympus BX41 microscope. For quantification of Bodipy staining, 18 images were collected for each condition, and Bodipy/ DAPI signal was calculated by Image J.

LDL aggregate analysis

Dil-LDL (1.25 μg/ml) was incubated with indicated peptides in PBS for 18 hours. Aggregate fluorescence was evaluated using the microscope. After LDL aggregate was washed by PBS, the aggregate was lysed by 1% SDS and Dil-signal was also quantified on Spectramax microplate reader (Molecular Biosystems) with at excitation 530 nm/ emission 580 nm excitation.

Lipoprotein uptake

Cells were treated with 1.25 μg/ml of pHrodo-LDL, 4.13 μg/ml of pHrodo-oxLDL, 0.25 μg/ml of pHrodo-VLDL or 1.25 μg/ml of pHrodo-HDL. After incubation at 37 °C for 18 hours, cells were washed by PBS and nuclei were counterstained with NucBlue™ Live ReadyProbes™ Reagent (Hoechst, Thermo Fisher Scientifics) for analysis with microscopy. Fluorescence was quantified on

the microplate reader after 18 hours unless otherwise specified. Fluorescence intensities were normalized by the concentration of lipoproteins added. For blocking of LDL uptake, pit stop (20 μM, Sigma Aldrich), genistein (100 μM, Sigma Aldrich), anti-LDLR antibody (R&D Systems, AF2148, 1:50 dilution), anti-SR-B1 antibody (Novus Bilogicals, NB400-134, 1:100 dilution) or anti-CD36 antibody (Thermo Fisher Scientific, MA5-14112, 3:100 dilution) was added 1 hour prior to pHrodo-LDL treatment. LDL uptake was evaluated after incubation for 2 hours when anti-LDLR antibody or anti-SR-B1 antibody was used for THP-1 cells, and after incubation for 4 hours when anti-LDLR antibody was used for human monocyte-derived macrophages (HMDMs).

LDL binding assay

Cells were incubated with 100 μg/ml of Dil-LDL at 4 °C for 20 minutes. After cells were washed by PBS, they were lysed with 1% SDS and fluorescence was quantified on the microplate reader.

Proximity ligation assay (PLA)

PLA was performed according to the manufacture's instruction. Briefly, cells were treated with LL37 and/ or native LDL or biotinylated LDL (12.5 μg/ml) for 30 minutes (native LDL) or 90 minutes (biotinylated LDL). The physical proximity of LDL and LDLR, SR-B1 or CD36, and LL37 and LDLR, SR-B1 or CD36 was determined with fluorescence-based PLA that produces a red fluorescent signal. Then, cells were fixed with 4 % paraformaldehyde for 10 minutes. Following fixation, blocking buffer (Sigma-Aldrich) was used to prevent nonspecific antibody binding, and cells were incubated with two primary antibodies for 18 hours at 4°C. The following antibodies were used with 1: 50 dilution: mouse anti-LDLR antibody (Santa Cruz Biotechnology, sc-18823), rabbit anti-LDLR antibody (Thermo Fisher Scientific, MA5-32075), mouse anti-CD36 antibody (Thermo Fisher Scientific, MA5-14112), rabbit anti-CD36 antibody (Thermo Fisher Scientific, MA5-32433), mouse anti-SR-B1 antibody (Santa Cruz Biotechnology, sc-518140),

rabbit anti-SR-B1 antibody (Novus Bilogicals, NB400-134), mouse anti-LL37 antibody (Santa Cruz Biotechnology, D-5), rabbit anti-Cramp antibody and mouse anti-biotin antibody (Santa Cruz Biotechnology, sc-101339). After wash with PBS, cells were further incubated with Plus and Minus oligonucleotide probe conjugated secondary antibodies, and further hybridization, ligation, amplification and detection of the PLA was performed using Duolink In Situ Detection Reagents Red (Sigma -Aldrich). PLA signals were captured on an Olympus BX41 microscope. For quantification, 6 images were collected for each condition in each experiment, and PLA signals per cell were calculated by image J.

Flow cytometric analysis

For evaluation of LDL uptake in vitro, THP-1 cells were treated with 1.25 μg/ml of pHrodo-LDL and LL37. Eighteen hours after that, cells were isolated by pipetting. For evaluation of LDL uptake in vivo, 1ml of 4% thioglycolate (VWR) was injected into mouse peritoneal cavity. Forty-eight hours after that, 1.25 μg/ml of pHrodo-LDL in 100uL of PBS was injected intraperitoneally. Eighteen hours after the injection of pHrodo-LDL, peritoneal cells were isolated. The isolated cells (THP-1 or mouse peritoneal cells) were incubated in a FACS staining buffer (PBS containing 5% BSA and 0.01% NaN3) with human Fc receptor binding inhibitor polyclonal antibody (eBioscience) or mouse CD16/32 antibody (eBioscience). Then, cells were stained with antibodies. The following antibodies were used with 1: 100 dilution: Alexa Fluor 700 anti-human CD45 (eBioscience, 56-9459-42), PE/Cy7 anti-mouse F4/80 (BioLegend, 123114), APC/Cy7 anti-mouse CD11b (TONBO bioscience, 25-0112-U100), Brilliant violet 711 anti-mouse CD45 (BioLegend, 103147). Dead peritoneal cells were stained by the addition of the Fixable Viability Dye eFluor 506 (eBioscience), and were gated out from the analysis. Flow cytometry was performed on the Biorad ZE5 machine, and the data were analyzed using FlowJo.

Ex vivo LDL uptake of endothelial cells

Aortas were collected from wild-type mice, and were split longitudinally to expose the endothelium. The tissues were then incubated with 7.5 µg/ ml of dil-LDL and LL37 in RPMI medium for 24 hours. Frozen sections (8 µm) were made to evaluate LDL uptake.

Co-immunoprecipitation and Immunoblot Analyses

Biotinylated LDL (3.1 µg/ml) was incubated with LL37 or Cramp. After 18 hours, biotinylated LDL was co-immunoprecipitated with rabbit anti-biotin antibody (Fortis Life Sciences, A150-109A, 1:20 dilution) or rabbit IgG isotype control (Thermo Fisher Scientific, 31235, 1:100 dilution) using Dynabeads™ Protein A Immunoprecipitation Kit (Thermo Fisher Scientific). To evaluate binding of LL37 to apolipoprotein B in serum, serum from *Apoe*$^{-/-}$ mice, LL37$^{tg/tg}$ *Apoe*$^{-/-}$ mice or human healthy blood donor was co-immunoprecipitated with mouse anti-LL37 antibody (Santa Cruz Biotechnology, D-5, 1:20 dilution) or mouse isotype IgG control (Thermo Fisher Scientific, 14-4714-85, 1:50 dilution) using Dynabeads™ Protein G Immunoprecipitation Kit (Thermo Fisher Scientific). The collected samples were eluted with 20 µL of elution buffer and 8 µL of the samples were loaded onto Novex™ 10 to 20%, Tricine, 1.0 mm, Mini Protein Gels (Thermo Fisher Scientific) or NuPAGE™ 3 to 8%, Tris-Acetate, 1.0–1.5 mm, Mini Protein Gels (Thermo Fisher Scientific), transferred to a polyvinylidene difluoride membrane, and probed with rabbit anti-LL37 antibody (1:500 dilution), rabbit anti-Cramp antibody (1:500 dilution) or rabbit anti-apolipoprotein B antibody (Thermo Fisher Scientific, ARC0920, 1:500 dilution or abcam, ab139401, 1:500 dilution). IRDye-conjugated anti-rabbit or anti-mouse secondary antibodies (Licor Bioscience, 1: 5000 dilution) were used. The images were acquired and quantitative analysis of signal intensity was performed on an Odyssey CLx Imaging System.

Small angle X-ray scattering (SAXS) experiments

LDL particles were incubated with LL37, LL34, and Cramp at a peptide-to-lipid (P/L) molar ratio of 3/35. SAXS experiments were conducted at Stanford Synchrotron Radiation Lightsource (SSRL, Beamline 4-2) using monochromatic X-rays of wavelength λ =1.378 Å (energy 9keV). A Pilatus3 X 1M detector (pixel size 172 µm) was used to collect the scattering signal. Multiple measurements were performed on each sample to ensure data quality. The two-dimensional (2D) powder diffraction pattern was integrated with the Nika package 1.81 in Igor Pro 9. The integrated intensity I(q) was plotted against the q, where q was the magnitude of the scattering vector defined as $q=(4\pi\sin\theta)/\lambda$ with θ the scattering angle and λ the wavelength of the X-rays. The form factor fitting was done using the SasView 5.0.6 (the core-shell ellipsoidal model) and the best fit in the lower range of q was obtained by adjusting the radius of the shell (r), the shell thickness (t), the ratio of the shell thickness at the pole to the equator (λ), the aspect ratio (ξ), and the relative electron density of the core with respect to the shell (ρ).

qRT-PCR

The RNA was isolated from the THP-1 cells or HMDMs using Purelink RNA isolation columns (ThermoFisher Scientific) according to the manufacturer's instructions. RNA was quantified using a Nanodrop spectrophotometer (ThermoFisher Scientific), and up to 1000 ng of RNA was reverse-transcribed using Verso cDNA Synthesis Kit (ThermoFisher Scientific). Quantitative real-time PCR reactions were run on a CFX96 real-time detection system (Bio-Rad) using gene-specific primers and SYBR green master mix (Biomiga Inc). PCR primers were synthesized by Integrated DNA Technologies and the sequences or catalog number are shown on Supplemental Table 2.

RNA-sequencing

RNA was extracted using a Purelink RNA mini kit (Life technologies, USA). Isolated RNA was submitted to the UCSD IGM Genomics Center for RNA-sequencing performed on a high-output

run V4 platform (Illumina, USA) with a single read 100 cycle runs. Data alignment was done on Partek flow software (Partek, USA) with Tophat2 (version 2.0.8). Group comparisons were carried out at cut-offs with false discovery rate (FDR) <0.01. Gene ontology (GO) analysis and prediction of transcription factors that regulate these genes was performed on those genes showing more than 1.5 fold decreased expression after treatment with LDL and LL37 compared with no treatment and decreased expression after treatment with LDL and LL37 compared with LDL or LL37 monotherapy. Gene ontology (GO) term analysis was performed on GO TERM FINDER (https://go.princeton.edu/cgi-bin/GOTermFinder). Enrichr (https://maayanlab.cloud/Enrichr/) was used to predict the transcription factors.

Isolation of peritoneal macrophages

Peritoneal lavage was harvested from wild type mice. Collected peritoneal cells were resuspended in RPMI medium, and cultured for 2 hours at 37 °C. Nonadherent cells were removed by gently washing by PBS, and adherent cells were used as peritoneal resident macrophages for analyses.

Evaluation of atherosclerotic plaques and lipid analyses

Mice were anesthetized with isoflurane, blood was collected for lipid analyses, and the vascular system was perfused with PBS followed by 4% paraformaldehyde by left ventricle puncture. The heart and whole aorta were isolated and placed in 4% paraformaldehyde for fixation overnight. The heart was embedded in OCT compound, and frozen sections (8 μm) of the aortic sinus were obtained. The sections of the aortic sinus were stained with freshly prepared Oil red O working solution for 20 minutes. Then, the sections were rinsed with PBS and counterstained with Mayer's hematoxylin. For en face analysis, the thoracic aorta was cleaned. Then, the aorta was opened longitudinally and pinned, and was stained with the Oil Red O working solution. Plaque areas and

total vessel areas were evaluated by morphometry of obtained images using Image J software. Plasma lipid profiles were examined by Lab Services of UC San Diego.

Fast protein liquid chromatography (FPLC)

Serum was separated by gel filtration FPLC using a GE Superose 6 10/300 GL column in 0.15 M sodium chloride containing 0.01 M disodium hydrogen phosphate and 0.2 mM ethylenediaminetetraacetic acid, pH 7.4. Fractions (0.5 mL) were collected (0.5 mL/min) and total cholesterol and triglyceride levels were determined using commercially available kits (Sekisui Diagnostics). To measure LL37 levels in the fractions, same volumes of the fraction were applied for immunoblot analyses with rabbit anti-LL37 antibody (1:500 dilution) and ELISA for LL37 (Hycult Biotech).

Immunohistochemistry

Tissue blocks were fixed in 4% paraformaldehyde, and 8-μm sections were subjected to staining. Sections were blocked with UltraCruz Blocking Reagent (Santa Cruz Biotechnology), followed by incubation with primary antibodies for 18 hours at 4°C. Sections were subsequently incubated with appropriate fluorochrome-conjugated secondary antibodies, and nuclei were counterstained with DAPI. The following antibodies were used: rat anti-mouse CD68 antibody (Bio-Rad, FA-11, 1: 100 dilution), Alexa Fluor 568 anti-Rat IgG (Thermo Fisher Scientific, A-11077, 1: 250 dilution), Alexa Fluor 488 anti-LL37 antibody (Santa Cruz Biotechnology, sc-166770 AF488, 1: 100 dilution). For Nile red staining, sections were stained with Nile red (Thermo Fisher Scientific) for 2 hours. For quantification of the proportion of LL37/ CD68 double stained areas within whole LL37 stained areas, 4 images of the plaque per mouse were collected, and the proportion was measured by Image J.
